# Metabolic Disturbance Induced by the Embryo Contributes to the Formation of Chalky Endosperm of a Notched-Belly Rice Mutant

**DOI:** 10.3389/fpls.2021.760597

**Published:** 2022-01-05

**Authors:** Yang Tao, Atta Mohi Ud Din, Lu An, Hao Chen, Ganghua Li, Yanfeng Ding, Zhenghui Liu

**Affiliations:** ^1^College of Agriculture, Nanjing Agricultural University, Nanjing, China; ^2^Collaborative Innovation Center for Modern Crop Production, Nanjing Agricultural University, Nanjing, China

**Keywords:** seed development, embryo–endosperm interaction, chalkiness, metabolome, transcriptome, rice physiology

## Abstract

Grain chalkiness is a key quality trait of the rice grain, whereas its underlying mechanism is still not thoroughly understood because of the complex genetic and environmental interactions. We identified a notched-belly (NB) mutant that has a notched-line on the belly of grains. The line dissects the endosperm into two distinct parts, the upper translucent part, and the bottom chalky part in the vicinity of the embryo. Using this mutant, our previous studies clued the negative influence of embryo on the biochemical makeup of the endosperm, suggesting the need for the in-depth study of the embryo effect on the metabolome of developing endosperm. This study continued to use the NB mutant to evolve a novel comparison method to clarify the role of embryo in the formation of a chalky endosperm. Grain samples of the wild-type (WT) and NB were harvested at 10, 20, and 30 days after fertilization (DAF), and then divided into subsamples of the embryo, the upper endosperm, and the bottom endosperm. Using non-targeted metabolomics and whole-genome RNA sequencing (RNA-seq), a nearly complete catalog of expressed metabolites and genes was generated. Results showed that the embryo impaired the storage of sucrose, amino acid, starch, and storage proteins in the bottom endosperm of NB by enhancing the expression of sugar, amino acids, and peptide transporters, and declining the expression of starch, prolamin, and glutelin synthesis-related genes. Importantly, the competitive advantage of the developing embryo in extracting the nutrients from the endosperm, transformed the bottom endosperm into an “exhaustive source” by diverting the carbon (C) and nitrogen (N) metabolism from synthetic storage to secondary pathways, resulting in impaired filling of the bottom endosperm and subsequently the formation of chalky tissue. In summary, this study reveals that embryo-induced metabolic shift in the endosperm is associated with the occurrence of grain chalkiness, which is of relevance to the development of high-quality rice by balancing the embryo–endosperm interaction.

## Introduction

High population pressure in rice-consuming regions always demands an increase in production, but the pursuit for a concomitant increase in grain quality is also increasing due to the enhanced quest for high-quality food in the developing countries like China (Bandyopadhyay et al., 2019; Song J. M. et al., 2019; Guo et al., 2020). Major quality traits of rice grain include the physical appearance, cooking, and sensory and nutritional properties. Among them, chalkiness, the opaque part of rice grain, has been set up as one of the main goals in rice breeding, for it affects not only the visual appearance quality but also the milling recovery of intact grains (Xi et al., 2014). In addition, high temperature occurring at the grain-filling stage has been reported as the most influential environmental factor inducing chalkiness, indicating that grain chalkiness would be a major threat to the rice industry in the scenario of global warming (Shi et al., 2017; Wada et al., 2019; Chang et al., 2021; Park et al., 2021; Yang et al., 2021). Therefore, it is urgent to elucidate the mechanisms responsible for chalkiness formation in rice grain.

Grain chalkiness is a complex trait governed by multiple genes and their interactions with the variable environments, as reflected by the slow advances in elucidating its genetic and physiological foundation. Though a large number of quantitative trait loci (QTLs) had been mapped, only fewer genes had been cloned and characterized (Liu et al., 2010; Sreenivasulu et al., 2015; Zhu et al., 2018; Xie et al., 2021). Carbon (C) or nitrogen (N) metabolism are the most important biochemical processes for grain filling, and their products, the starch, and storage proteins form the foundation of rice quality. Previously, incomplete accumulation of starch was widely accepted as the distinct histochemical property of the chalky endosperm tissue (Lisle et al., 2000; Singh et al., 2003). Later, a microscopic observation revealed that the chalky tissue is loosely packed with protein bodies as well as starch granules, indicating the importance of a balance between C and N metabolism when dealing with grain chalkiness (Xi et al., 2014). Concerning the physiological mechanisms, mounting evidences show that within the endosperm, disturbance either in C or N metabolism can result in chalky tissue occurrence (Yamakawa et al., 2007; Yamakawa and Hakata, 2010; Lin et al., 2017a,b; Wada et al., 2019). The C and N metabolisms are interdependent, and they interact across various levels in plant metabolism, indicating that they have to be subtly balanced to produce a proper proportion of starch and proteins and thereby avoid the formation of chalkiness. It should be noted that most of the previous studies on grain chalkiness were centered on the physiological aspects within the endosperm. On the other hand, fewer studies have been conducted to clarify the mechanism of the formation of chalkiness from the perspective of exogenous factors like the embryo.

Rice grain contains three distinct components including diploid embryo, triploid endosperm, and diploid maternal tissues. Growing evidence in rice, *Arabidopsis*, and maize suggest the importance of a communication between these compartments (Nowack et al., 2006; Widiez et al., 2017; Song W. et al., 2019; Zheng et al., 2019; Doll et al., 2020a,b; Xiong et al., 2021). The endosperm supports and nurtures the developing embryo and in return, the embryo transmits the signal to allocate the nutrients and thus influence the biochemical makeup of the endosperm (Zheng et al., 2019; Doll et al., 2020a; Song et al., 2021; Zhang et al., 2021). It is therefore suggested that embryo–endosperm interaction and their mutual metabolic signaling influence the rice quality like grain chalkiness.

We identified a notched-belly (NB) rice mutant that has a dichotomous appearance of endosperm, with opaque tissue in the lower basal parts, proximate to the embryo, and the translucent appearance in the upper part of the endosperm (Lin et al., 2014). Using this mutant, we explored the mechanism of chalkiness formation through biochemical, proteomic, and transcriptomic analysis. Notably, the embryo exhibited a substantial influence on the biochemical makeup of the endosperm and had a substantial negative impact on the overall storage of proteins, amino acids, and minerals in the chalky endosperm (Lin et al., 2016). Such modifications in the endosperm composition were mainly associated with the changes in the expression levels of the genes involved in the regulation of metabolites transporters and hormonal signaling in the chalky endosperm (Lin et al., 2017a). Altogether, these findings suggested that the embryo is involved in the formation of grain chalkiness by allocating nutrient distribution to the endosperm.

Metabolomics is a powerful tool to narrow the knowledge gap between the genotype and phenotype by monitoring and sensing the metabolic state in plant cells under various conditions. The advancement in mass spectrometry has made it more efficient and convenient to explore the metabolic dynamics and the underlying signaling mechanisms for seed development (Yamakawa and Hakata, 2010; Matsuda et al., 2012; Lin et al., 2017b; Li et al., 2019; Chen et al., 2020). In addition, it is applicable to breeding programs because of the link between sensory traits and metabolic profiles (Oikawa et al., 2008). Formerly, we performed an untargeted metabolic analysis of the NB mutant, and found that the chalkiness formation was associated with a metabolic shift from primary C and N metabolism to secondary metabolism like reactive oxygen species scavenging, osmoregulation, and cell wall synthesis, thus extending the understanding of the metabolomic mechanism underlying grain chalkiness (Lin et al., 2017b).

Considering that the recent studies focus highly on the endosperm, with no consideration of the role of embryo–endosperm interaction, we continued to use the NB mutant to explore the physiological foundation of grain chalkiness, by comparing the metabolite accumulation and gene expression profiles of the developing embryo and endosperm. Furthermore, a novel comparison method was invented to interpret the role of embryo in regulating the metabolic process in the endosperm, with the following objectives: (i) uncovering the metabolic dynamics of endosperm and embryo; (ii) manifesting and quantifying the embryo effect on endosperm development; and (iii) elucidating the mechanism of chalkiness formation from the perspective of embryo–endosperm interaction. The findings obtained here could offer insights into the metabolic processes of grain formation, thus providing a valuable resource for the manipulation of molecular and physiological processes responsible for rice quality.

## Materials and Methods

### Plant Materials and Sampling

**FIGURE 1:**
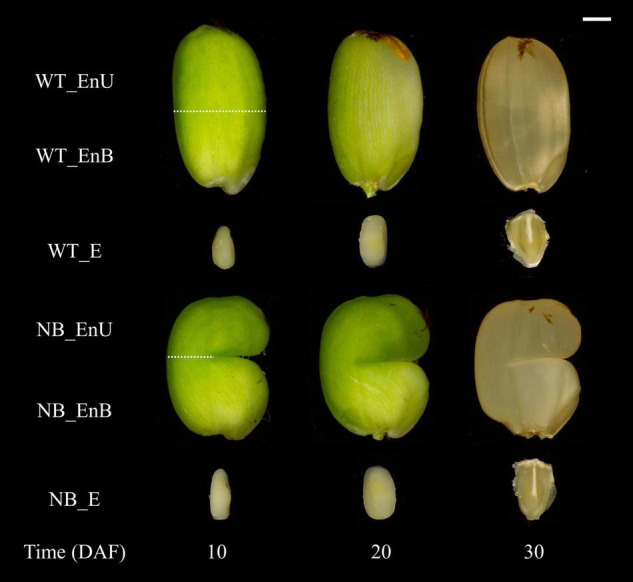
Overview of the time series samples of embryo and endosperms. Dotted line indicates the manual dissection of endosperm, cutting it into upper and bottom endosperms. DAF, days after fertilization; E, embryo; En, endosperm; EnB, bottom part of endosperm; EnU, the upper part of endosperm; NB, notched-belly rice mutant; WT, wild-type of Wuyujing-3. Bar = 1 mm.

The notched-belly mutant was obtained by treating the wild-type (WT) Wuyujing-3 with the mutagenic compound, ethyl methanesulfonate (EMS) (Lin et al., 2014). The experiment was conducted in 2018; rice seedlings were transplanted into a plastic pot of 29 cm in height and 30 cm in diameter. The pot was filled with 10 kg clay loam that has a pH of 6.41, 17.20 g kg^–1^ of organic matter, 0.95 g kg^–1^ of total N, 0.55 g kg^–1^ of total P, 11.79 g kg^–1^ of total K, 20.44 mg kg^–1^ of Olsen-P, and 91.10 mg kg^–1^ of exchangeable K. The plants were grown under natural environment condition at the vegetative stage. To ensure the consistency and stability of the environment during seed development, we translocated the plants with an identical flowering date into a growth chamber 2 days before anthesis. The growth conditions were kept similar to those mentioned by Lin et al. (2017b), with the modifications of day and night temperature to 31 and 24°C, respectively. The light intensity and the relative humidity were 600 μmol photons m^–2^ s^–1^ and 70 ± 5%, respectively. Middle rachis with a higher ratio of white-belly grains was sampled in three biological repeats for the NB and WT, at 10, 20, and 30 days after fertilization (DAF). Samples were immediately frozen by liquid nitrogen and stored at -80°C. For metabolomic and RNA-seq analysis, the developing grains were dehusked on the dry ice, and then manually dissected by the scalpel into two parts, the embryo and the endosperm. Subsequently, the endosperm was cut into the upper part (defined as the upper part of the endosperm, EnU) and the bottom part (EnB) along the notched line. For the WT, we divided the endosperm into two parts along the midline of the endosperm (Figure 1). It is to be noted that all the subsamples contained the outer layers of the grains, such as the seed coat and pericarp, due to technical difficulty.

### Metabolite Profiling by Multiple Analytical Platforms

Six biological replicates were used for metabolic analysis. An automated, robotic system MicroLab STAR (Hamilton, Reno, NV, United States) was used to prepare the embryo and endosperm samples for metabolite profiling. To ensure the quality, recovery standards were added for the quality check. The extraction was carried out with methanol by a vigorous shaking of 2 min. Subsequent centrifugation helped to recover the individual metabolites, and the evaporator was used to remove the organic solvent. Before the metabolomic investigation, the extracted samples were kept in nitrogen overnight. The resulting extract was divided into five fractions: two for analysis by two separate reverse phases (RP)/UPLC-MS/MS methods using positive ion mode electrospray ionization (ESI), one for analysis by RP/UPLC-MS/MS using the negative ion mode ESI, another for analysis by HILIC/UPLC-MS/MS using the negative ion mode ESI, and one was reserved for backup.

### Data Analysis and Construction of the Metabolic Atlas

The raw data from the three analytical platforms were extracted and processed in Metabolon Laboratory Information Management System. A reference library was created through approximately 1,500 authentic standards in Metabolon and used as a reference to identify the metabolites by an automated comparison. The quantification and data normalization of the significantly changing metabolites were performed according to previously used methods (Lawton et al., 2008; Rao et al., 2014). A brief data normalization step was employed to correct any instrument-based variation due to tuning differences across experimental days. The quantitative values of the metabolites were obtained from the counts of the integrated raw detector mass spectrometers. To preserve the variation with the data, all compounds from the extensively variable raw data were normalized directly on an identical graphical scale, with the normalized intensities scaled by their median values for each compound. Tests of significance were performed by Welch’s two-sample *t*-test using the R program. The database of Plant Metabolic Network (PMN^1^) was used to target the metabolic pathways. The metabolic atlas was constructed through the Kyoto Encyclopedia of Genes and Genomes) pathway (KEGG^2^). Principal component analysis (PCA) was performed to classify the data from 108 samples, using Omicshare tools.^3^

### Gene Expression Profiling Using RNA Sequencing

Three biological replicates were used for RNA sequencing (RNA-seq) analysis. About 0.1 g tissue sample was used to extract the RNA by a kit from Invitrogen (Carlsbad, CA, United States). The extract was diluted with 100 μL of RNAase-free water. The RNA concentration was quantified by Nanodrop, with its quality evaluated using LabChip from Agilent (Santa Clara, CA, United States). Two evaluation systems, namely Agilent 2100 Bioanalyzer and StepOnePlus Real-Time PCR System (Applied Biosystems, Waltham, MA, United States) were used for the qualification and quantification of the sample library. Nibbonbare (IRGSP-1.0^4^) and BGISEQ-500 (BGI, Shenzhen, China) were referenced for reading and mapping analysis, and library sequencing, respectively. Only clean-read was processed further by HISAT2 (V2.1.0) (Kim et al., 2015) to ensure effective and high-quality mapping (Chen et al., 2018). Eventually, the expression level of different genes was estimated using RSEM (V1.2.8) software (Li and Dewey, 2011). A gene is considered as expressed only if its value of fragments per kilobase of transcript per million mapped reads (FPKM) is either equal to or exceeds 1. Notably, genes with an absolute value of log_2_ ratio ≥1 compared with an FDR corrected value of *P* ≤ 0.001 were designated as differentially expressed genes (DEGs) (Wang et al., 2010).

### Validation of Transcriptome Data Using Quantitative Reverse Transcription-PCR

For the credibility of RNA-seq data, it was verified by the quantitative reverse transcription-PCR (qRT-PCR). Twelve genes that are involved in C and N metabolism were selected for the verification of their expression pattern. Primers were designed through Primer 5.0 (An et al., 2009), as in Supplementary Table 1. Comparison of both the results revealed a similar pattern, verifying the validity of the RNA-seq data (Supplementary Figure 1).

### Metabolites and Genes Coexpression and Functional Enrichment Analysis

The coexpression analysis to study the ontology of metabolites and genes in the embryo and the endosperm was carried out using the MeV (Howe et al., 2010). Values of *Z*-score were used as input for MeV, and clustered by using the *K*-means and Pearson’s correlation coefficients among the metabolites and genes. Metabolites were enriched in metabolic pathways using MBROLE online enrichment software (V2.0^5^) (López-Ibáñez et al., 2016). The *P*-value was used to identify the significant KEGG categories (*P*-value < 0.05). The annotation of genes into functional categories was carried out using the KEGG annotation. The hyper function in R software (R Core Team, 2013) was used to perform the functional enrichment of genes, with a default setting.

### Starch and Protein Content Analysis

For starch content analysis, the samples were subjected to ethanol extraction, followed by digestion in perchloric acid. Contents of starch were quantified by using an anthrone agent and measuring the absorbance at 620 nm (Hansen and Møller, 1975). The fractions of storage proteins including albumin, globulin, prolamin, and glutelin were extracted and measured according to the method of Ning et al. (2010).

### Statistical Analysis

The concentration data of the starch and proteins in the study are averages of triplicate observations. The SPSS statistics package version V19.0 (SPSS Inc., Chicago, IL, United States) was used for the statistical analysis, and Duncan’s multiple range test (*P*-value < 0.05) was used for multiple comparisons for the significant difference.

## Results

### General Description of the Metabolomics in the Developing Embryo and Endosperm

**FIGURE 2:**
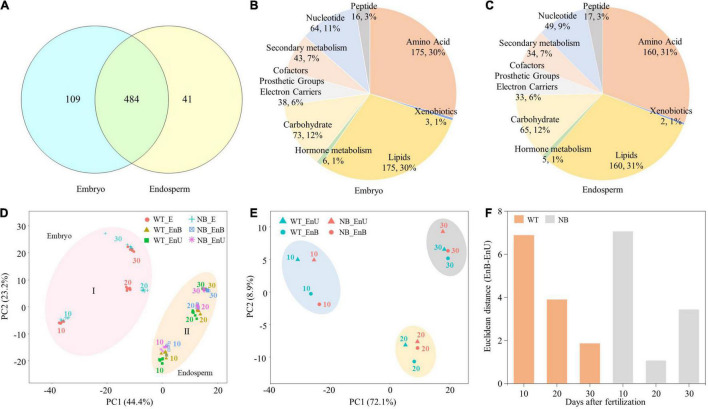
Summary of metabolome data of embryo and endosperm. **(A)** Venn diagram of the 634 metabolites accumulated in the embryo and endosperm. **(B,C)** Classification and distribution of the identified metabolites in the developing embryo **(B)** and endosperm **(C)**. **(D)** PCA of the seed tissue metabolites populations. PCA plot shows two distinct groups of embryo and endosperm metabolites populations: Group I for embryo and Group II for endosperm. **(E)** PCA showing global metabolome relationships of time-series samples from the upper part and bottom part of the endosperm. **(F)** The distance between the upper part and the bottom part of the endosperm in PCA at each stage. Numbers of 10, 20, and 30 represent the sampling time points (days after fertilization, DOF).

A total of 634 distinctly annotated metabolites were identified from all samples. Among them, 484 metabolites were accumulated in both the embryo and the endosperm, while only 109 and 41 metabolites were specifically accumulated in the embryo and the endosperm, respectively (Figure 2A). The metabolites identified in the embryo mainly covered the central metabolism pathway and partially the secondary metabolism pathways, including 175 amino acids, 73 carbohydrates, 175 lipids, 38 CPGEC (cofactors, prosthetic groups, and electron carriers), 64 nucleotides, 16 peptides, 43 secondary metabolites, 6 phytohormones, and 3 xenobiotics (Figure 2B). Within the endosperm, the identified metabolites consisted of 160 amino acids, 65 carbohydrates, 160 lipids, 33 CPGEC, 49 nucleotides, 17 peptides, 34 secondary metabolites, 5 phytohormones, and 2 xenobiotics (Figure 2C). As a whole, the general mapping of metabolites revealed almost a similar division of metabolites in the embryo and the endosperm.

However, further classification by PCA analysis shows that the identified metabolites and genes can be divided into two primary groups (the first component), each being associated with the embryo (I) and the endosperm (II), respectively (Figure 2D). The second component of PCA discriminated the early, middle, and late stages of development within the embryo and the endosperm (Figure 2D). Such a categorization indicated that the accumulation of metabolites during rice seed development is both tissue- and stage-specific (Figure 2D). Subsequently, to uncover the correlation between the upper and bottom endosperms across the developmental stages, we carried out another PCA that classified the metabolome data of two endosperm parts (Figure 2E and Supplementary Figure 2). Results showed that the pattern of metabolomic development of endosperm parts varied between WT and NB. For instance, the difference between the upper endosperm and the bottom endosperm decreased linearly along with the seed development for the WT. On the contrary, NB showed a “V-type” pattern regarding this difference, being the largest at 10 DAF, the lowest at 20 DAF, and recovered at 30 DAF (Figure 2F). Collectively, the PCA analysis clearly bifurcated the metabolites across the developmental stages and suggested a disturbance in the developmental process of the bottom endosperm of NB due to its proximity to the embryo.

### Coexpressed Metabolite Sets of Embryo and Endosperm Development

**FIGURE 3:**
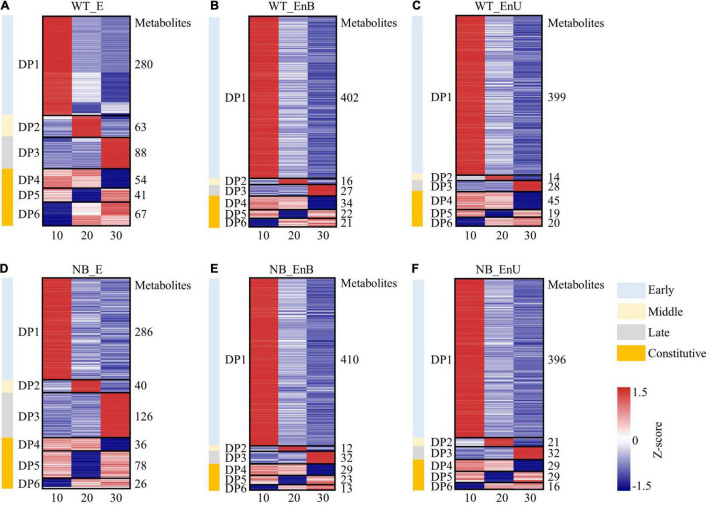
Metabolite dynamics of the developing embryo and endosperm for WT **(A–C)** and NB **(D–E)**. Dynamic patterns of metabolites in different coexpression modules are shown for the embryo **(A,D)**, the bottom part of the endosperm **(B,E)**, and the upper part of the endosperm **(C,F)**. Expression data were *Z*-score standardized from –1.5 to 1.5; the number of metabolites in each module is shown on the right. Numbers of 10, 20, and 30 represent sampling time points (days after fertilization, DOF).

**FIGURE 4:**
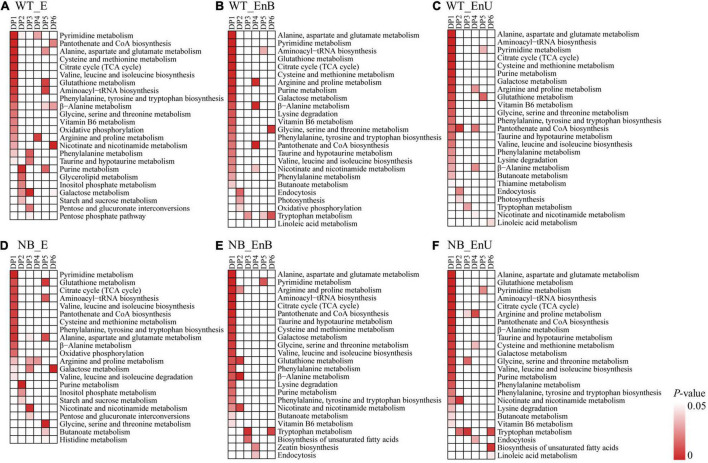
Kyoto Encyclopedia of Genes and Genomes (KEGG) enriched in different coexpression modules of embryo and endosperm for WT **(A–C)** and NB **(D–F)**. Only significant categories (*P*-value < 0.05) are displayed.

To gain further insight into the metabolic changes during the rice seed development, we divided all tissue-annotated metabolites into six clusters based on their accumulation patterns using the *K*-means clustering algorithm (Figure 3). We identified the metabolites of three modules (DP4–DP6) that were enriched at more than one stage in the embryo and the endosperm, respectively, indicating some common cellular metabolic processes across several stages. In addition, clustering also helped to map out the compounds whose contents were more prevalent at one out of the three developmental stages (DP1–DP3), indicating the specific function of these modules at the corresponding stage (Figure 3). The coexpression clustering as well as the KEGG enrichment classification, both verified the PCA grouping and confirmed the stage and tissue specificity of the metabolites within the embryo and the endosperm (Figures 3, 4). Notably, the amount and species of metabolites in the embryo and the endosperm were similar between the two genotypes (Supplementary Table 2). Therefore, we took WT as an example to describe the metabolic dynamics of the developing embryo and endosperm.

### Metabolic Processes in the Developing Embryo

Cellular metabolic processes characterizing each developmental stage were identified by KEGG terms that were overrepresented at particular coexpression modules (Figures 3A, 4A). The early-stage was best represented by the module, dominant pattern-1 (DP1), as typified by the overrepresentation of the KEGG terms for the citrate cycle, oxidative phosphorylation, and amino acid metabolism. This is consistent with the high requirement of energy during embryo formation. The middle stage featured the module, DP2 that was enriched in KEGG terms including starch and sucrose metabolism, inositol phosphate metabolism, and galactose metabolism, which coincides with the maturation of the embryo. The DP3 presented an upregulation of metabolites involved in phenylalanine metabolism, and taurine and hypotaurine metabolism, suggesting the dormancy of the embryo. In addition, the metabolites formed modules, DP4–DP6 accumulated broadly in the embryo across the sample time points, involving amino acid metabolism, purine and pyrimidine metabolism, and nicotinate and nicotinamide metabolism.

### Metabolic Processes in the Developing Endosperm

Compared with the embryo, the majority of the metabolites decreased during endosperm development. This may be due to the gradual transformation of C and N metabolites into starch, protein, and lipid (Figures 3B,C, 4B,C). Metabolites involved in the citrate cycle and in the metabolism of amino acid, glutathione (GSH), purine, and pyrimidine were enriched in the early stage (DP1). This may be related to the cell differentiation of endosperm. The proportion of metabolites in the middle stage (DP2) was the least, and the KEGG terms of endocytosis were enriched, which may coincide with the desiccation and program cell death of endosperm cells. The content of metabolites associated with endosperm maturation and those involved in the tryptophan metabolism increased significantly in the late stage (DP3). Notably, metabolites responsible for arginine and proline metabolism, nicotinate and nicotinamide metabolism, pantothenic acid and coenzyme A biosynthesis, and linoleic acid metabolism were overrepresented in DP4–DP6 and broadly expressed throughout the development.

### A Novel Method to Quantify the Effect of Embryo on Endosperm Development

**FIGURE 5:**
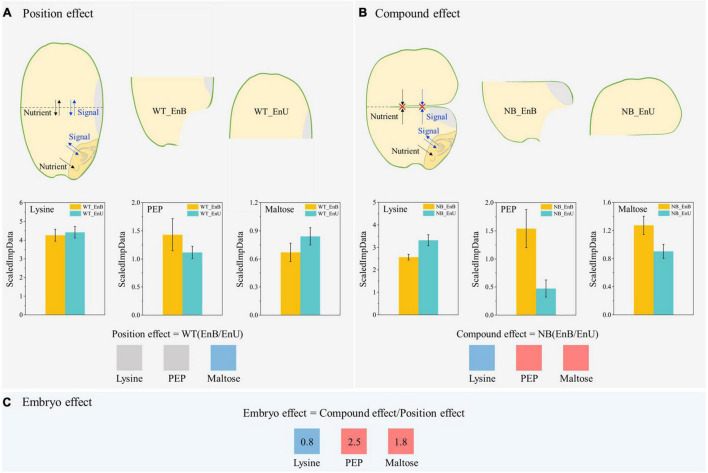
Overview of the new comparison method for quantification of the embryo effect on endosperm development. This method comprises three key components: **(A)** Position effect: it is estimated by the comparison of upper endosperm (WT_EnU) and bottom endosperm (WT_EnB) of wild-type (WT), indicating the difference in two endosperm parts. Note that within this grain, both nutrients and signals could easily travel between the upper and bottom parts. **(B)** Compound effect: it is estimated by the comparison of the bottom (NB_EnB) and upper (NB_EnU) endosperm of the NB, showing the combined effect of embryo and position. Within the grain of NB, the notched line severely cut-off the nutrient supply and the signal movement to the upper endosperm. As a result, the major impact of embryo is trapped inside the proximate part of the endosperm (NB_EnB). **(C)** Embryo effect: after quantifying both the position and compound effects, we can roughly estimate the effect of embryo on endosperm, by the comparison of NB_(EnB/EnU)_/WT_(EnB/EnU)_. Three metabolites are used as examples to demonstrate the working principle of this method, as explained in detail in the main text. Three metabolites are used as examples to demonstrate the working principle of this method, as explained in detail in the main text. Red, navy blue, and gray boxes indicate upregulation and downregulation, and no marked differences of lysine, phosphoenolpyruvate, and maltose in the bottom endosperm as affected by position, compound, and embryo effect, respectively. For comparisons of position and compound effect, a marked difference is recorded according to Duncan’s multiple range test (*P* < 0.05). For comparisons of embryo effect, a marked difference is defined as those ≤0.95 (downregulation) or ≥1.05 (upregulation). The numbers in boxes of panel **(C)** represent the calculated value for the embryo effect. PEP, phosphoenolpyruvate.

The marked difference between the NB and WT in the metabolomic profiling and its changing pattern across the developmental stages indicated that the embryo could have a substantial influence on the metabolic processes in the endosperm (Figures 2E,F). Therefore, to quantify the embryo effect, we utilized the NB mutant to develop a novel comparison system, by comparing the upper and bottom endosperms of the NB, with those of the WT as reference (Figure 5).

Briefly, the comparison system employed in the present study comprises three components. (1) Position effect: it indicates the difference in two parts of the endosperm based on their position within the WT grain, where both the nutrients and the signals can easily travel between their upper endosperms (WT_EnU) and bottom endosperms (WT_EnB) (Figure 5A). (2) Compound effect: comparison between the bottom (NB_EnB) and the upper (NB_EnU) endosperm of the NB mutant shows the combined effect of embryo and position (Figure 5B). Notably, the notched lines severely cut off the nutrient supply and signal movement to the upper endosperm. As a result, the influence of embryo is trapped inside the proximate part of the endosperm (NB_EnB). (3) Embryo effect: eventually, after quantifying both the position and compound effects, we can roughly evaluate the effect of embryo on the endosperm, by eliminating the position effect from the compound effect (Figure 5C).

To clearly explain the working principle of the comparison method, we employed three key metabolites, lysine, phosphoenolpyruvate (PEP), and maltose, which are involved in amino acid metabolism, energy metabolism, and starch metabolism, respectively (Figure 5). First, the position effect significantly downregulates the maltose content, but has no marked effect on those of the lysine and PEP. In contrast, the compound effect of the position and embryo increases the PEP and maltose whereas it reduces lysine. Finally, when the position effect is expunged, it uncovers the promotion of maltose and PEP and the inhibition of lysine under the embryo effect. Taken together, the metabolomic anomaly observed within the seed tissues of WT and NB during the metabolomic profiling was eventually verified by utilizing the novel comparison method. In addition, it also verified that the “V-shaped” pattern observed between the upper and bottom endosperm was caused by the altered metabolism under the embryo effect. Eventually, the influence of embryo on endosperm metabolism was confirmed.

### Embryo Effect on the Metabolic Dynamics of the Developing Endosperm

After verifying the metabolic disturbance in endosperm under the embryo effect, we further used the proposed comparison method to uncover the detailed consequences of the metabolic alteration in the endosperm and the relevant underlying mechanisms. By performing the complementary analysis of the metabolic and gene expression profiling, we not only assured that the embryo can disturb the metabolic processes in the endosperm, but also identified some key pathways in the endosperm that are induced by the embryo. Importantly, the identified pathways have clear relevance to the formation of chalky tissue, suggesting the embryo effect as one of the causes behind the chalky belly in the NB mutant. These pathways are explained in detail in the next section.

### The Ability of Metabolites Transport in the Bottom Chalky Endosperm Was Enhanced by the Embryo

**FIGURE 6:**
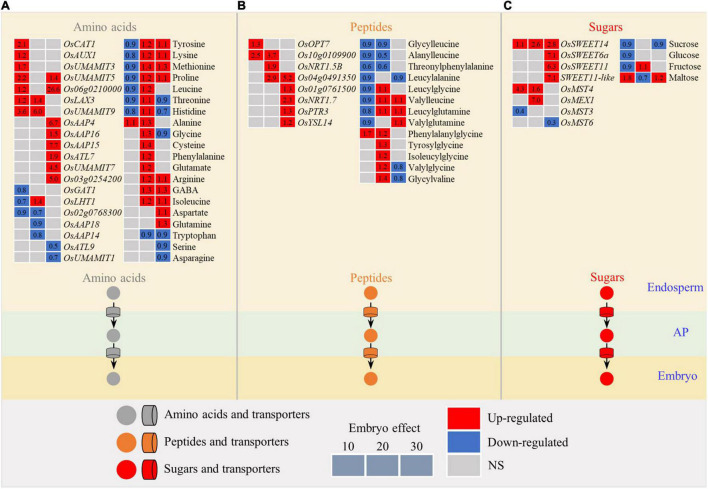
Expression patterns of metabolites and genes involved in the transport of amino acids **(A)**, peptides **(B)**, and sugars **(C)** in the chalky endosperm as affected by the embryo. Gray, red, and navy-blue boxes denote non-significant, significant upregulation, and significant downregulation by the embryo, respectively. Data in boxes are the calculated values of the embryo effect. Numbers of 10, 20, and 30 above the gray boxes represent the sampling time points (days after fertilization, DOF). AP, apoplasm; NS, non-significant.

The embryo had a significant influence on the expression pattern of transporters for amino acids, peptides, and sugars, resulting in the differential accumulation of relevant substrates (Figure 6). In general, most transporters were enhanced in the endosperm by developing the embryo at the early stage (10 DAF) with the exceptions of three amino acid transporters, such as *OsGAT1*, *OsLHT1*, and *Os02g0768300* (Figure 6A), and one sugar transporter *OsMST3* that were downregulated (Figure 6C). This increased expression of transporters in the endosperm was accompanied by a decline in the relevant substrates, such as amino acids (tyrosine, lysine, methionine, proline, leucine, threonine, and histidine; Figure 6A), peptides (glycylleucine, alanylleucine, threonylphenylalanine, leucylalanine, leucylglycine, valylleucine, leucylglutamine, and valylglutamine; Figure 6B), and sugars (sucrose, glucose (Glu), and fructose; Figure 6C). Overall, the upregulated trend of the transporters suggested the excavation of nutrients from the endosperm, likely due to the developing embryo, that acted as a “sink” within the developing seed at the early stage of embryo development.

At the middle and the late stages (20 and 30 DAF), most of the transporter genes still showed higher activity (Figure 6). However, the content of the substrates showed an opposite trend relative to that at 10 DAF. For example, with the exception of threonine, histidine, glycine, tyrosine, serine, and asparagine, the contents of other amino acids increased significantly (Figure 6A). Likewise, except glycylleucine, alanylleucine, threonylphenylalanine, and leucylalanine, the other nine dipeptides showed an overall upward trend (Figure 6B). In addition, fructose markedly increased at 20 DAF, but sucrose and Glu did not change (Figure 6C). At 20 DAF, the embryo enters the dormancy stage, not competing for nutrients with the endosperm. Nutrients, such as amino acids, peptides, and sugars, still continue to input into the bottom endosperm for accumulating storages, resulting in the simultaneous increase in the amino acids and peptides and their transporters. This may partly explain the inconsistency of the changing pattern of the nutrients between the early stage and the middle and late stages of embryo development.

### Catabolism of Starch in the Bottom Chalky Endosperm Was Induced by the Embryo

**FIGURE 7:**
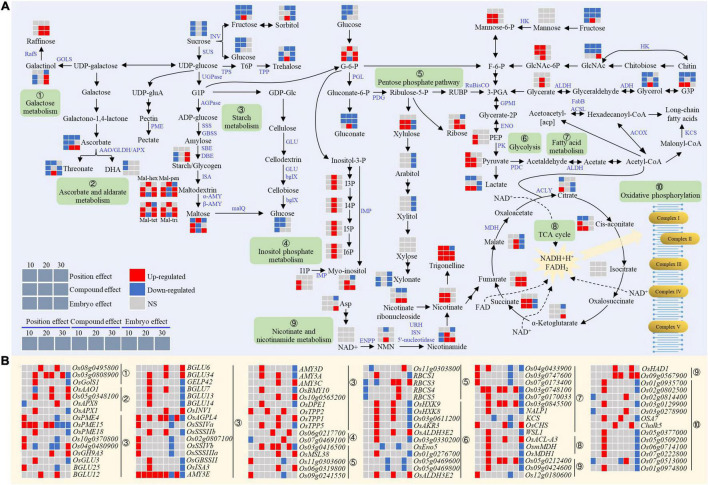
Expression patterns of metabolites **(A)** and genes **(B)** for carbohydrates metabolism in the chalky endosperm as affected by the embryo. Gray, red, and navy-blue boxes denote non-significant, significant upregulation, and significant downregulation by the position, compound, and embryo effect, respectively. Numbers of 10, 20, and 30 above the gray boxes represent the sampling time points (days after fertilization, DOF).

Genes and metabolites involved in carbohydrate metabolism were differentially expressed under the influence of the developing embryo (Figure 7). At the early stage (10 DAF), the embryo had a negative effect on sucrose, Glu, and fructose levels in the endosperm, probably due to its nutrient consumption (Figure 7A). Notably, despite the upregulation of *OsTPP2* gene that is related to the trehalose biosynthesis, the content of this sugar utilization related to disaccharide, decreased simultaneously with the other important sugars (Figure 7). Consequently, the insufficiency of the sucrose resulted in the decreased activity of starch synthesis genes, including *OsAGPL4*, *OsSSSIVa*, *OsSSIVb*, *OsSSSIIIa*, and *Os02g0807100* (Figure 7B). Meanwhile, maltose, the product of starch breakdown, and its precursors (Mal-hex, Mal-pen, Mal-tet, and Mal-tri) showed a significant increase, implying an accelerated breakdown of starch (Figure 7A). Similarly, *AMY3E*, an α-amylase gene for starch catabolism, also demonstrated a decreasing trend at 10 DAF (Figure 7B). Collectively, the deficiency of sucrose and the enhancement of starch degradation led to the reduction in starch content. Conversely, at the middle stage (20 DAF), sucrose metabolism and starch synthesis were enhanced in the endosperm, as evidenced by the increased accumulation of starch and the decrease of maltose and its precursor. Meanwhile, fructose, the decomposition product of sucrose, increased at 20 DAF and trehalose also showed the same trend (Figure 7A). In addition, the starch synthesis gene (*OsAGPL4*) and the degradation gene (*AMY3E*) were upregulated and downregulated, respectively (Figure 7B). Altogether, the metabolic pattern suggested that the developing embryo at the early stage induced the starch catabolism which might cause the impaired endosperm filling.

### Carbon Metabolism Is Shifted to Secondary Metabolisms in the Chalky Endosperm by the Embryo

The embryo had a positive effect on the inositol phosphate metabolism (Figure 7). At the early stage, myo-inositol and I1P were mainly accumulated, while during the later stages of endosperm development, phytic acid (I6P) and its precursors I5P, I4P, and I3P were significantly overrepresented in the chalky endosperm (Figure 7A). For example, I1P was significantly increased at 10 DAF, and myo-inositol and I6P and its precursors were upregulated at 20 or 30 DAF. In addition, the myo-inositol synthesis genes, *Os07g0469100* and *Os06g0217700* also showed the same trend (Figure 7B).

Ascorbate significantly decreased in the early stage but increased in the later stage (Figure 7A). Similarly, its downstream product, threonate also decreased at 10 DAF. In the metabolic pathway of ascorbate, AAO, GLDH, and APX participate in the synthesis of dehydroascorbic acid (DHA). The expression of *OsAAO1*, *GLDH* (*Os05g0348100*), and *OsAPX8* genes increased at 10 DAF or 30 DAF, while *OsAPX1* significantly decreased at 30 DAF (Figure 7B). However, there was no significant change in the DHA content during endosperm development (Figure 7A).

The content of raffinose increased significantly at the 20 and 30 DAF, while its precursor galactinol, first decreased at 10 DAF, and then increased at 30 DAF (Figure 7A). Galactinol synthase (GOLS) and raffinose synthase (RafS) catalyze the biosynthesis of raffinose (Falavigna et al., 2018; Li et al., 2020). *OsGolS1* was downregulated at 30 DAF, while the expressions of two RafS genes (*Os08g0495800* and *Os03g0808900*) increased at 10 DAF and 20 DAF, respectively (Figure 7B). In addition, the contents of other osmotic substances, such as erythritol, mannitol, and trigonelline, showed an increasing trend in the 20 DAF (Figure 7A).

Taken together, these trends of C metabolism clearly suggested the diversion of metabolic activities from the central storage-based metabolism to other secondary metabolic pathways that might limit the nutritional storage capacity of the endosperm, resulting in chalkiness formation.

### N Metabolism Switched From Storage Proteins Biosynthesis to Secondary Metabolism in the Chalky Endosperm by the Embryo

**FIGURE 8:**
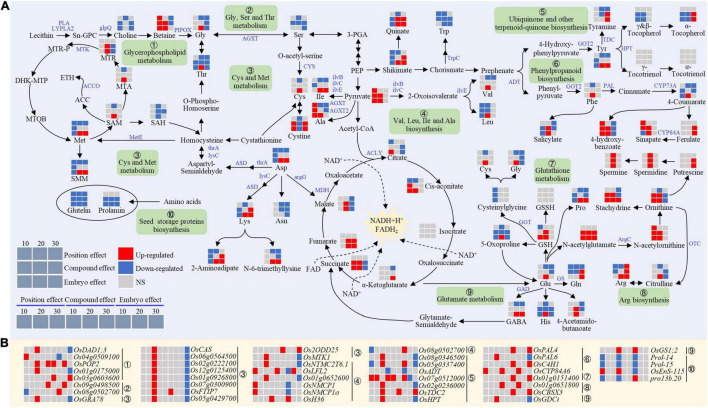
Expression patterns of genes **(A)** and metabolites **(B)** for amino acids metabolism in the chalky endosperm as affected by the embryo. Gray, red, and navy-blue boxes denote non-significant, significant upregulation, and significant downregulation by the position, compound, and embryo effect, respectively. Numbers of 10, 20, and 30 above the gray boxes represent the sampling time points (days after fertilization, DOF).

At the early stage, the embryo had a negative effect on amino acid levels in the endosperm (Figure 8A). The decrease of amino acids in the endosperm was accompanied by a decline in the expression levels of prolamin (*Prol-14* and *Prol-15*) and glutelin (*OsEnS-115*) synthesis genes (Figure 8B), which in turn inhibited the biosynthesis ability of storage proteins and resulted in a lower content of prolamin and glutelin (Figure 8A).

Arginine (Arg) metabolism in the chalky endosperm was markedly affected by the embryo (Figure 8). At the early and middle stages, ornithine, citrulline, and Arg showed an increasing trend (Figure 8A). The synthesis of Arg was first induced by the acetylation of Glu to produce *N*-acetylglutamate, which caused a subsequent increase of N-acetylornithine (NAO), ornithine, citrulline, and Arg. Moreover, in the arginine metabolism process, ArgC (*Os01g0651800*) and OTC (*OsCBSX3*) responsible for generating NAO and citrulline, exhibited upward trends at the early and late stages (Figure 8B). In addition, the increase of ornithine content in the endosperm was accompanied by the increased stachydrine, an important osmotic regulator in the plant cell. Betaine, another key osmoprotectant, showed a similar trend with stachydrine (Figure 8A).

Another remarkable effect of the embryo was detected in the glutamate metabolism. The GSH showed an increasing trend at the early and middle stages, which may be attributed to the altered glutamate metabolism (Figure 8A). For example, Glu content had no change at the early stage, but the contents of its derivatives (histidine and proline) decreased, indicating that the metabolic process was tilted toward the GSH synthesis pathway. In addition, the Glu level was upregulated at the middle stage, which in return enhanced the accumulation of GSH, histidine, and proline (Figure 8A).

In summary, it was observed that similar to C metabolism, the embryo also influenced the N metabolism of the NB endosperm, limiting the storage ability of endosperm and causing chalkiness at the bottom part of the endosperm.

## Discussion

Embryo–endosperm interaction considerably affects the seed development and consequently the physico-chemical properties of rice grain, such as the chalkiness. This study took advantage of the NB mutant to devise a novel comparison method for quantifying the impact of embryo on metabolic processes in the developing endosperm, unraveling the fact that the embryo has a noticeable effect of diverting C and N metabolism from the biosynthesis of storage compounds to secondary pathways in the chalky endosperm. This finding reveals new aspects on the role of embryo in the formation process of rice quality, and helps to map out effective strategies for high-quality rice breeding.

### Metabolic Signatures for Embryo and Endosperm Development

Metabolomics, combined with other “omics” methodologies, has emerged as an effective method for the studies of functional genomics and systems biology (Ghatak et al., 2018). Rice seed is a delicate biological system, mainly consisting of two genetically distinct tissues, the diploid embryo, and triploid endosperm (Wu et al., 2016a). So far, only few studies have studied both the embryo and endosperm in the same experiment (Galland et al., 2017). In the present study, using non-targeted metabolomics, we represented the distinct metabolic details about the embryo and the endosperm during seed development. The results revealed that both the embryo and endosperm samples were well distinguished by PCA, demonstrating a significant difference in the metabolism between two compartments. By *K*-means clustering algorithm and KEGG enrichment analysis, we integrated the cellular metabolic processes across the development phases of embryo and endosperm. Metabolic signatures of the embryo and endosperm at the major stages of seed developments are detailed as follows.

At the early stage (10 DAF), most of the morphogenetic events in the embryo have already occurred at this stage (Itoh et al., 2005), and the endosperm has finished tissue differentiation, forming two subregions, aleurone, and starchy endosperm, and starts storing starch and proteins (Wu et al., 2016b). At this phase, the metabolites related to glycolysis, TCA cycle, and oxidative phosphorylation were overrepresented both in the embryo and endosperm, indicating that the two compartments share common metabolic processes, which is consistent with the report by Belmonte et al. (2013).

At the middle stage (20 DAF), the embryo greatly grows to its maximum volume and starts to mature (Itoh et al., 2005). Meanwhile, the galactose, starch, sucrose, and inositol phosphate metabolism were active, which may play an important role in the transformation of embryo development from organ formation to maturity. For example, phytic acid (inositol 6-phosphate, I6P) is the principal storage form of phosphorus (P) in cereal grains and is considered a key compound in inositol phosphate metabolism (Perera et al., 2018). Previously, the phytic acid accumulation was observed during the rice seed development which was enhanced particularly at the middle stage (20 DAF) (Lin et al., 2017b). Similarly, in the present study, the rapid accumulation of phytic acid was observed, which can be taken as a sign of embryo maturation. Simultaneously, the endosperm starts to store the nutrients at the maximum rate. Heat map display of all data showed a clear overall decline in the relative levels of the vast majority of compounds as the seeds matured (Figure 3). This pattern is more obvious at this stage as C and N start to incorporate into macromolecules (starch, proteins, and lipids), which was in line with the finding of Lin et al. (2017b). In addition, the metabolites and genes related to starch and sucrose metabolism were also overrepresented (Supplementary Figures 3, 4).

At the late stage (30 DAF), the embryo becomes tolerant of desiccation after completion of reserve accumulation and undergoes a developmentally programmed dehydration event leading to dormancy and a quiescent state (Manfre et al., 2009). The starchy endosperm cells die completely upon seed maturation and desiccation (Zeeman, 2015). Low internal oxygen levels may be advantageous during seed desiccation by alleviating the oxidative damage of membranes and enzymes, which would otherwise lead to defects in seed vitality and longevity (Angelovici et al., 2010). In this study, genes related to peroxisome were commonly enriched in both the embryo and the endosperm at this stage, indicating that the antioxidant process is essential for seed desiccation.

### Effect of Embryo on Metabolic Transition of Developing Endosperm

As a major source of human calories, the rice seed is mainly composed of the two fertilization products, embryo and endosperm, and their surrounding maternal tissues. The formation of a viable rice seed requires a close cooperation between the embryo and endosperm (Lafon-Placette and Köhler, 2014; Robert, 2019; Anderson et al., 2021; Xiong et al., 2021). Knowledge of the embryo–endosperm interaction is essential for dissecting the mechanisms responsible for resource allocation within the developing seed, which is critical for the formation of both grain yield and quality. In comparison to another important cereal crop of maize (*Zea mays*), fewer studies have been conducted on this bidirectional communication in rice seeds (An et al., 2020). Maize is an excellent cereal model for research on the interaction between two compartments because of the relatively larger size of the embryo that accounts for 20% of the grain volume (Nagasawa et al., 2013; Chen et al., 2014). In contrast, the embryo tissue makes up only 2–3% of rice grain on the base of dry matter (Juliano and Tuaño, 2019), which may be a limitation for in-depth analysis of this interaction (Lafon-Placette and Köhler, 2014).

As interpreted above, the use of NB mutant made an interesting finding that the embryo has a role in regulating the accumulation of starch and protein in the chalky endosperm (Lin et al., 2016). Based on this, the current study continued to use the NB and WT as materials, thereby designing a new comparison system that shows the effect of the embryo on endosperm development. Our results found that the embryo impairs the storage of sucrose, amino acid, starch, and storage proteins in the bottom endosperm of NB by enhancing the expression of sugar, amino acid, and peptide transporters and declining the expression of starch, prolamin, and glutelin synthesis-related genes. The results were consistent with the previous studies and confirmed the transporters as an important “switch” to regulate nutrients allocation between the embryo and the endosperm (Lin et al., 2017a,b). Furthermore, the competitive advantage of the developing embryo in translocating nutrients from the endosperm transforms the bottom endosperm into a “superior source” by shifting the C and N metabolism from the anabolism of storages to other metabolic pathways, such as central metabolism and osmotic and antioxidant regulation, verifying the speculation that embryo exerts a “counter-acting regulation force” on the endosperm during the seed development by influencing its metabolic processes and gene expression (An et al., 2020). However, considering its insignificant proportion in the rice grain, the significant impact of the mini-size embryo on the endosperm development remains to be further verified.

### Mechanism Underlying Chalkiness Formation From the Perspective of Embryo–Endosperm Interaction

Grain chalkiness is highly undesirable for the rice industry because of its negative effect on the appearance quality as well as the sensory and milling quality of rice. Though a number of chalkiness-related QTLs were mapped, only few genes were isolated and functionally characterized, with the formation and regulatory mechanism of rice chalkiness still being one the most formidable challenges for rice geneticists (Liu et al., 2010; Sreenivasulu et al., 2015; Zhu et al., 2018; Xie et al., 2021). From the viewpoint of crop physiology, the hampered work on the genetic dissection of grain chalkiness may be associated with the intricate nature of the physiological aspects of chalkiness formation. As explained in section “Introduction,” the occurrence of chalky tissue is a result of unbalanced accumulation of starch and protein. Therefore, any disturbance in C or N metabolism can cause the formation of this opaque tissue due to the loose packing of starch granules or/and protein bodies. As shown in the current study, compared with the upper translucent endosperm, the levels of sucrose, amino acids, starch, and storage proteins in the bottom chalky endosperm are all downregulated. This finding agrees with our previous study (Lin et al., 2016, 2017a,b), supporting the critical role of C and N metabolism in chalkiness formation. However, most of the related studies are confined to the endosperm itself; fewer studies have been conducted to examine the role of the embryo. As a result, the signals that regulate the physiological processes in the endosperm are still unclear.

In this study, we successfully applied a novel comparison method to show the direct effect of embryos on the metabolic processes of the endosperm. Using WT as a reference, a comparison of the bottom and upper parts of NB grains confirmed the findings of our previous work as well as identified new clues responsible for grain chalkiness formation. Our results found that the embryo impairs the storage of sucrose, amino acid, starch, and storage proteins in the bottom endosperm of NB, by enhancing the expression of sugar, amino acid, and peptide transporters and declining the expression of starch, prolamin, and glutelin synthesis-related genes. In particular, the embryo effect was more evident at the critical stage of chalkiness formation (10 DAF). This stage is critical both for embryo morphogenesis and endosperm development, as evidenced by the highly expressed *Chalk5* gene that is responsible for chalkiness formation (Li et al., 2014). Furthermore, taking advantage of the altruistic behavior of endosperm toward embryo development, the developing embryo attempted to deplete its “adjacent source,” the endosperm, by the excessive excavation of nutrients. And this could alter the endosperm metabolism, causing chalkiness formation due to the poor accumulation of storage compounds. In the previous study, the source-limitation was considered a key reason for chalkiness formation, reflected by the insufficiency of nutrients from photosynthetic organs, such as leaves that begins to decrease in the biosynthesis ability of storage compounds (starch and proteins) in the endosperm (Wang et al., 2008; Hu et al., 2020). In the present study, we uncovered the mechanism of chalky endosperm formation from the perspective of the embryo–endosperm interaction, showing that the chalky endosperm formation is not only caused by source limitation, but also by nutrient competition between the embryo and the endosperm. This finding indicates that the interaction between the embryo and the endosperm plays an important role in the formation of rice quality.

## Conclusion

This study unraveled the metabolic signatures across the development phases of the embryo and the endosperm, and demonstrated a direct effect of the embryo on the transition of metabolic patterns in the endosperm. A novel comparison method based on the NB mutant revealed a substantial influence of the embryo on the metabolic processes in the endosperm. The embryo imposed an inhibitory effect on the accumulation of sucrose, amino acid, starch, and storage protein in the endosperm, which might be related to the increased expression of sugar, amino acid, and peptide transporters and decreased the expression of starch, prolamin, and glutelin synthesis genes. Importantly, the embryo–endosperm interaction, particularly, the competitive advantage of the embryo in extracting the nutrients from the endosperm during the embryo development, transformed the C and N metabolism of the endosperm from storage biosynthesis to secondary metabolic pathways. Consequently, the impaired endosperm filling resulted in the formation of chalkiness. In summary, this integrative study highlights the role of the embryo in the endosperm chalkiness formation, with the underlying metabolic processes being identified. Hopefully, the results obtained here should help rice breeders to work out new pipelines for nurturing high-quality rice cultivars. Future studies should be centered on the genetic control of the reprogramming of metabolic pathways in the developing endosperm, and clarify the molecular mechanism involved in the communication between the embryo and the endosperm.

## Data Availability Statement

The original contributions presented in the study are publicly available. This data can be found here: https://www.ncbi.nlm.nih.gov/bioproject/PRJNA722833.

## Author Contributions

YT had the main responsibility for data collection and analysis. YT and AM wrote the manuscript. LA, HC, GL, and YD revised the manuscript. ZL had the overall responsibility for experimental design, project management, and manuscript preparation. All authors contributed to the article and approved the submitted version.

## Conflict of Interest

The authors declare that the research was conducted in the absence of any commercial or financial relationships that could be construed as a potential conflict of interest.

## Publisher’s Note

All claims expressed in this article are solely those of the authors and do not necessarily represent those of their affiliated organizations, or those of the publisher, the editors and the reviewers. Any product that may be evaluated in this article, or claim that may be made by its manufacturer, is not guaranteed or endorsed by the publisher.

## Footnotes

1
http://www.plantcyc.org/

2
http://www.genome.jp/kegg/pathway.html

3
https://www.omicshare.com

4
https://www.ncbi.nlm.nih.gov/genome/?term=IRGSP-1.0

5
http://csbg.cnb.csic.es/mbrole2/analysis.php
