# Supplementary material for: Metabolic Disturbance Induced by the Embryo Contributes to the Formation of Chalky Endosperm of a Notched-Belly Rice Mutant
Source: Front Plant Sci. 2022 Jan 5;12:760597. doi: 10.3389/fpls.2021.760597 (PMC8767064; doi:10.3389/fpls.2021.760597)
Supplement: Supplementary file 1 [file Table_1.DOCX]

Table S1. Details of primers used in this study

| Gene ID |  | Gene description | F-Primer | R-Primer |
| --- | --- | --- | --- | --- |
| Os08g0345800 |  | *OsAGPS2b* | TGAAGGGTTCGTTGAAGTCC | GTCCATGCGGTAAAGGTGAT |
| Os05g0187100 |  | *OsHXK7* | AGGACATAATGCCGTGTTCAA | GTCCAGACCTTGCTTCTCCAT |
| Os08g0473600 |  | *AMY3E* | GCGGGTGGTACAACTTTCTG | AGCGGTGGTTGATGACGAT |
| Os08g0113100 |  | *OsFKII* | TGATGATGAGTTCGGGCAC | GAGATGGAGCCGTAGTGGAA |
| Os01g0730300 |  | *OsTPS3* | GGGCCAAGGAGAAAGAGTATGT | CATTGCCTCTGCTGTTGTATCA |
| Os10g0450900 |  | *OsGRP-2* | GCTCTTGCCATCCTTGTCCT | CACTACCACCTCCCTCACCAT |
| Os11g0508600 |  | *OsSWEET14* | CGCATCGTGGTTCTTGGTT | ACGTTGGGAAGAGCGACATAT |
| Os05g0331800 |  | *Prol-14* | TTTCGCCTTTGCTCTTCTTACT | TGTTGCCAGACTTGGTTGTTT |
| Os02g0306401 |  | *OsNAAT1* | GGGCTGGGAGATTGACCTTA | CCACCTTGGATAAATGCTCGT |
| Os01g0111600 |  | *OsMFT2* | GCAACGACCTCTACACCCTG | CCCGGTATGTTAACCACGAT |
| Os01g0374000 |  | *GSTF12* | TACCAATGCCTCATCCTCCC | TACCTGAACTTGCACAGACGC |
| Os04g0413500 |  | *OsGIF1* | GGTTCGTTGACACGGACATC | GAGCTGCGACACCTTGATCT |
