# Supplementary material for: Metabolic Disturbance Induced by the Embryo Contributes to the Formation of Chalky Endosperm of a Notched-Belly Rice Mutant
Source: Front Plant Sci. 2022 Jan 5;12:760597. doi: 10.3389/fpls.2021.760597 (PMC8767064; doi:10.3389/fpls.2021.760597)
Supplement: Supplementary file 2 [file Table_2.DOCX]

Table S2. Numbers of the metabolites identified in the embryo and endosperm of WT and NB.

| Super pathway | WT | | | NB | | |
| --- | --- | --- | --- | --- | --- | --- |
|  | E | EnB | EnU | E | EnB | EnU |
| Lipids | 175 | 159 | 160 | 174 | 159 | 160 |
| Carbohydrate | 73 | 65 | 65 | 73 | 65 | 65 |
| Amino Acid | 175 | 160 | 160 | 175 | 160 | 160 |
| Nucleotide | 38 | 33 | 33 | 38 | 33 | 33 |
| Secondary metabolism | 64 | 49 | 49 | 64 | 49 | 49 |
| Cofactors, Prosthetic Groups, Electron Carriers | 16 | 17 | 17 | 16 | 17 | 17 |
| Hormone metabolism | 6 | 5 | 5 | 6 | 5 | 5 |
| Peptide | 43 | 32 | 34 | 43 | 29 | 32 |
| Xenobiotics | 3 | 2 | 2 | 3 | 2 | 2 |
| Total | 593 | 522 | 525 | 592 | 519 | 523 |
