# Supplementary figures and images for: Metabolic Disturbance Induced by the Embryo Contributes to the Formation of Chalky Endosperm of a Notched-Belly Rice Mutant
Source: Front Plant Sci. 2022 Jan 5;12:760597. doi: 10.3389/fpls.2021.760597 (PMC8767064; doi:10.3389/fpls.2021.760597)

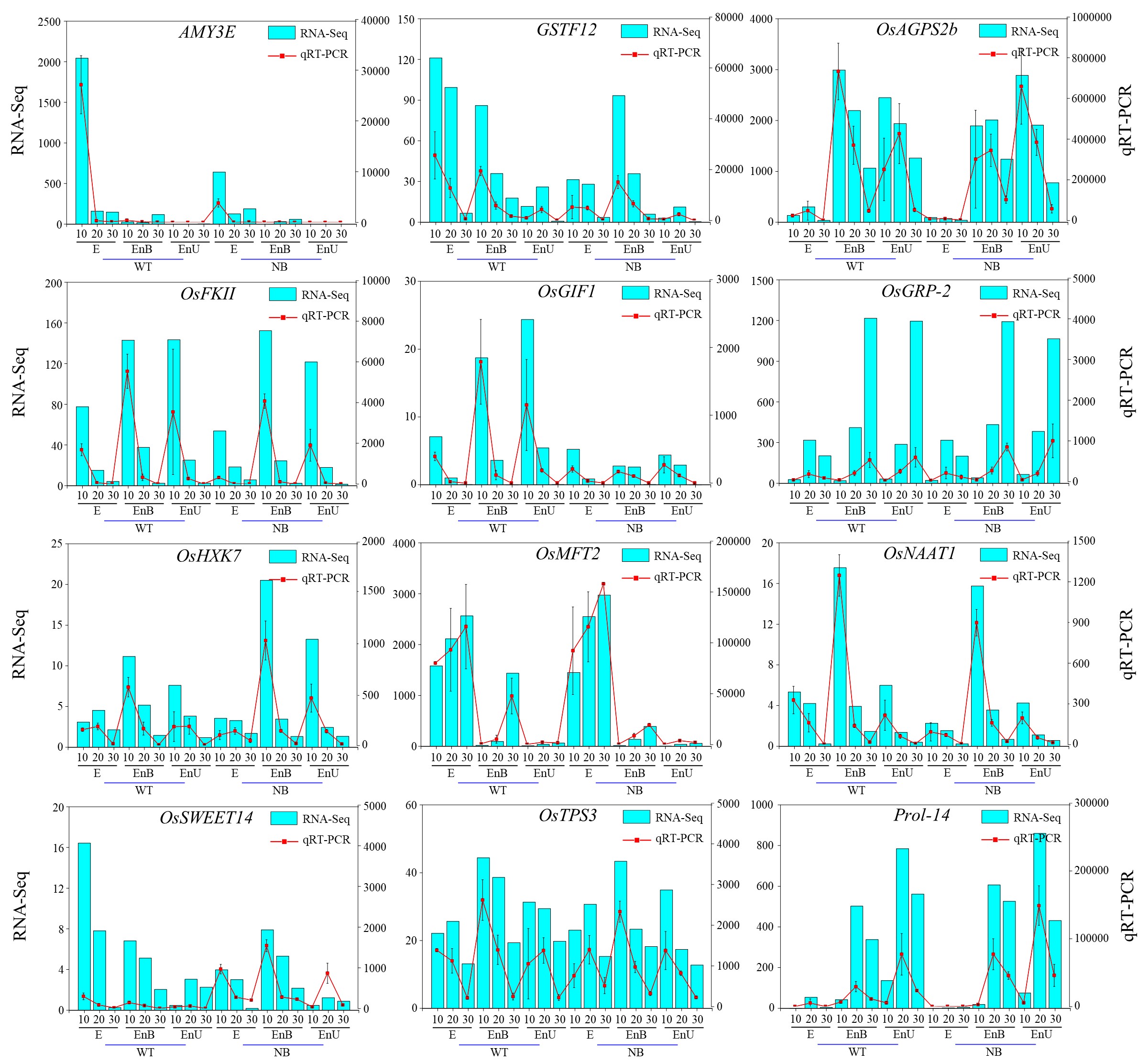

Supplement: Supplementary file 3 [file Image_1.JPEG]

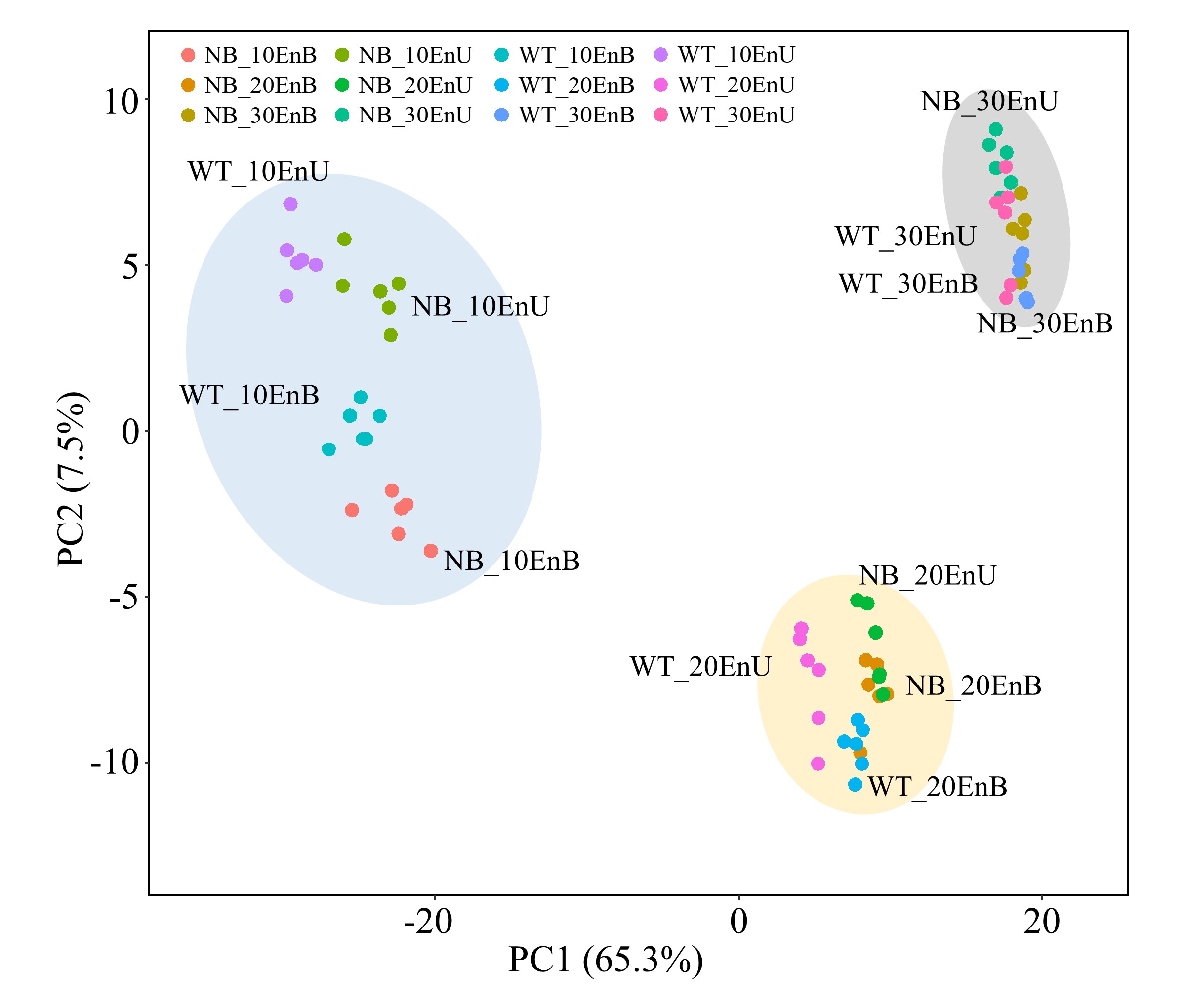

Supplement: Supplementary file 4 [file Image_2.JPEG]

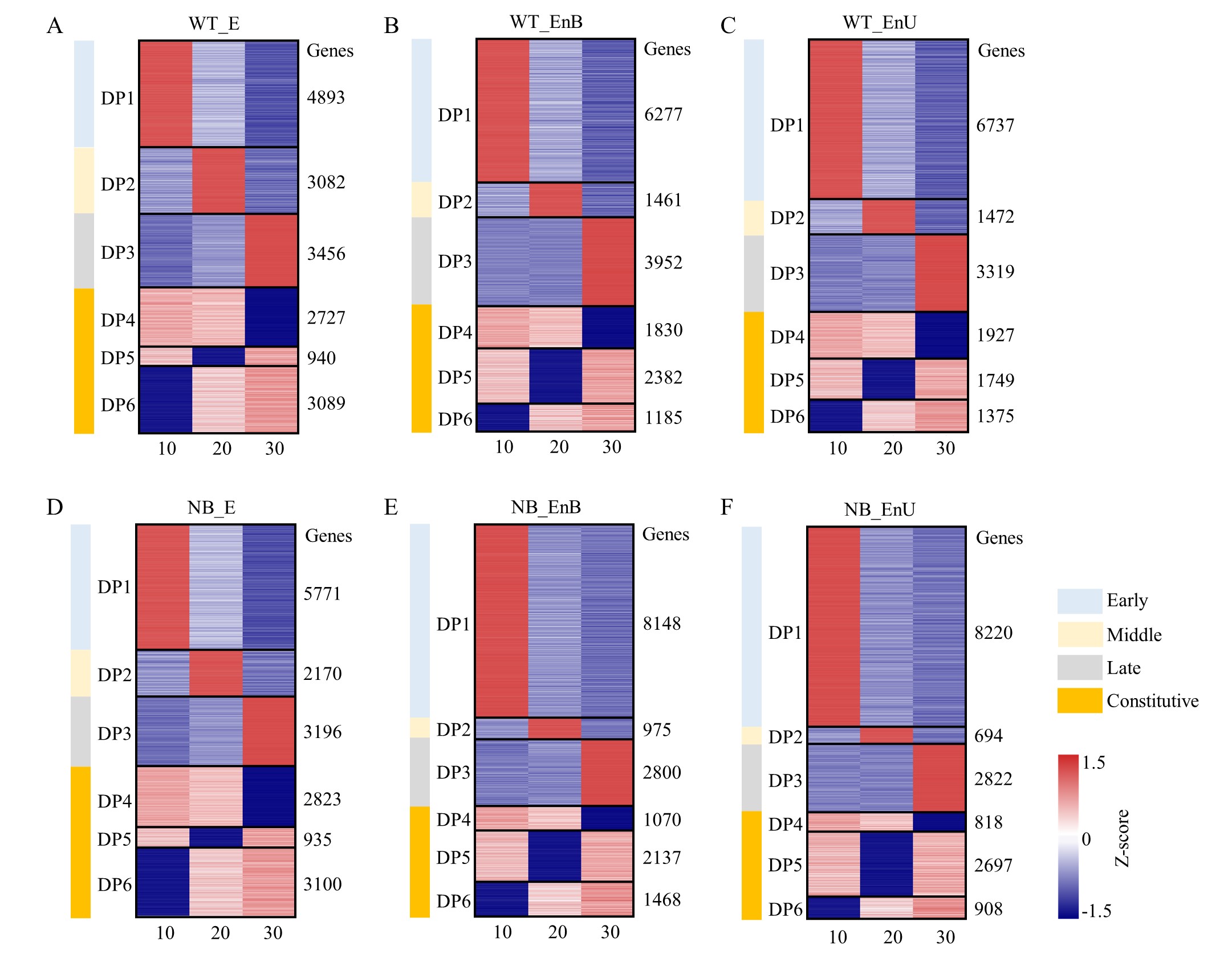

Supplement: Supplementary file 5 [file Image_3.JPEG]

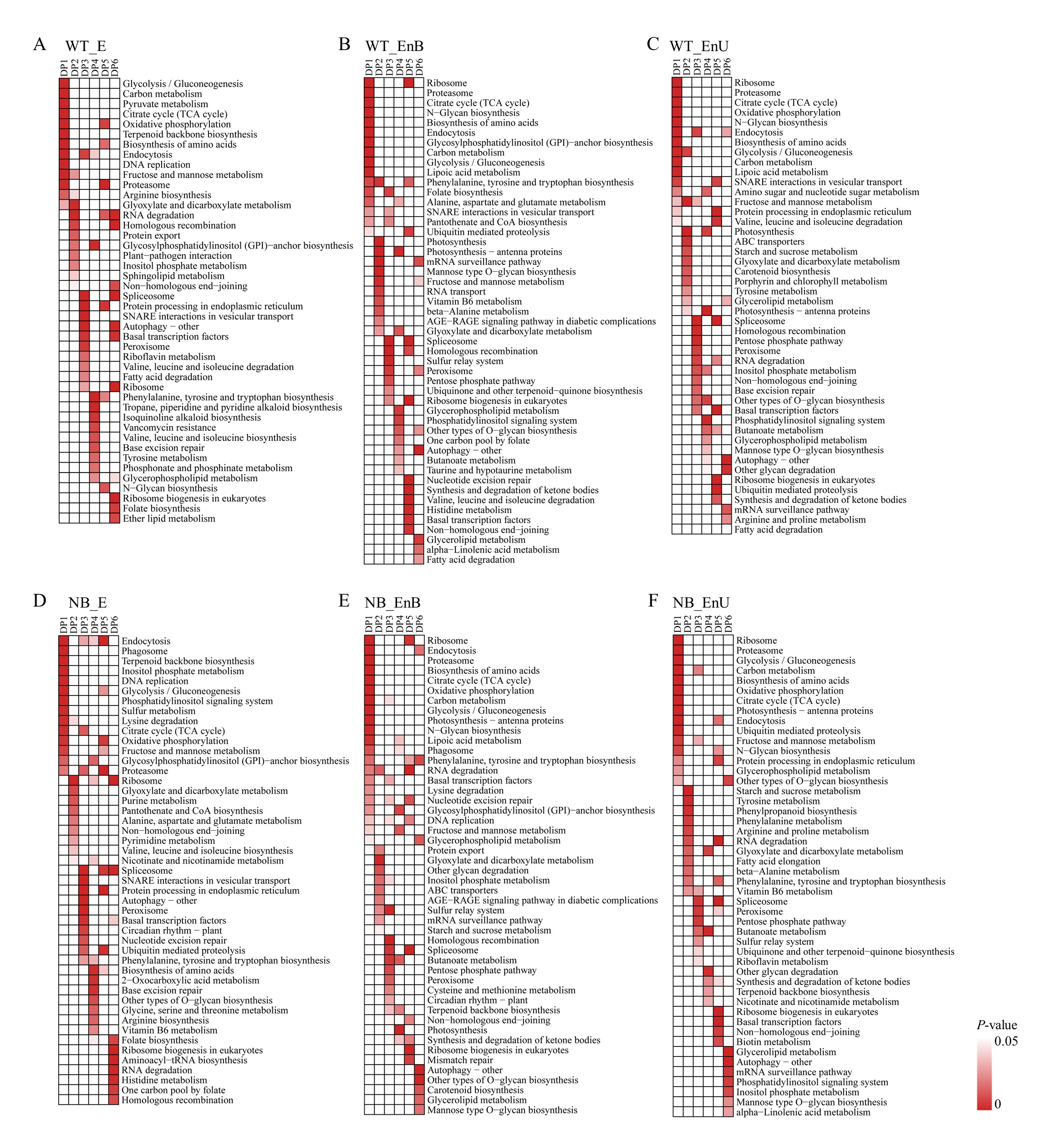

Supplement: Supplementary file 6 [file Image_4.JPEG]
